# Gut Microbiome Differences Between Early Alzheimer Disease and Idiopathic Normal Pressure Hydrocephalus

**DOI:** 10.1097/WAD.0000000000000726

**Published:** 2026-05-28

**Authors:** Emilia Brandt, Anne Koivisto, Pedro Pereira, Ella Mustanoja, Petri Auvinen, Toni Saari, Minna Rusanen, Ville Leinonen, Filip Scheperjans, Virve Kärkkäinen

**Affiliations:** * Department of Neurology; ‡‡ Department of Neurosurgery, Institute of Clinical Medicine, School of Medicine, University of Eastern Finland; † Department of NeuroCenter, Kuopio University Hospital, Kuopio; ‡ Department of Neurosciences, Clinicum, Faculty of Medicine; # Institute of Biotechnology; ** Institute for Molecular Medicine Finland (FIMM), Helsinki Institute of Life Sciences; ¶ Clinicum, University of Helsinki; § Department of Geriatrics; ∥ Department of Neurology, Helsinki University Hospital, Helsinki; †† Geriatric Center, Wellbeing services county of North Karelia, Joensuu, Finland

**Keywords:** Alzheimer disease, gastrointestinal microbiome, hydrocephalus, normal pressure, neurodegenerative diseases

## Abstract

**Background:**

Alzheimer disease (AD) and idiopathic normal pressure hydrocephalus (iNPH) are neurodegenerative diseases causing memory decline. Previous studies have demonstrated an altered gut microbiome (GM) in both conditions. In this study, we compared the GM composition between the groups to find out how if the GM composition differed between the cognitively healthy individuals (CO) and AD groups, as well as between the AD and iNPH groups.

**Methods:**

Thirty-seven CO participants, 21 mild AD patients and 10 participants with shunted iNPH gave fecal samples, which were subjected to 16S amplicon sequencing. Then, genus-level differences were analyzed. Information about comorbidities and diet was collected, and cognitive function was evaluated.

**Results:**

Compared with the CO group, *Anaerovorax* and an unknown genus of the Comamonadaceae family increased, whereas *Enterobacter, Absicoccus, Buttiauxella, Raoultella*, and *Lacticaseibacillus* decreased in the AD group. Compared with the iNPH group, *Paramuribaculum*, an unknown genus of the Desulfovibrionaceae family, *Ruficoccus* and *Mitsuokella* increased, whereas *Anaeromassilibacillus* and *Desulfovibrio* decreased in the AD group.

**Conclusions:**

We demonstrated differences in the GM composition between the AD and CO groups, as well as between the AD and iNPH groups. To our knowledge, this is the first report to compare the 2 neurodegenerative diseases and demonstrate GM differences.

Increasing research links neurological diseases with disturbances in the gastrointestinal (GI) system, particularly the gut microbiome (GM). The bidirectional pathway known as the gut-brain axis involves neuronal pathways as well as metabolic and neurotransmitter-mediated signaling.^1^ The bacteria of the GM produce the majority of the metabolic activity in the GI system and play a crucial role in host immunology and overall health.^2,3^

The GM composition may provide us with insight into the pathophysiology of various diseases and reveal novel biomarker candidates. GM alterations are robustly established in Parkinson disease (PD) and have already led to the first therapeutic clinical trials.^4–6^

In a previous study, we demonstrated that the GM composition is altered in iNPH patients compared with cognitively healthy individuals.^7^ Furthermore, emerging research suggests GM changes in early AD.^8^

Dementia, the loss of cognitive abilities, such as memory and executive functions, is increasing globally, with Alzheimer disease (AD) being the primary cause.^9^ AD typically progresses asymptomatically for a few decades, and as the neuronal loss worsens, it leads to increasing cognitive decline, dementia, and ultimately death.^10^

Neuropathological manifestations of AD include beta-amyloid accumulation and hyperphosphorylated tau-protein aggregations, which can be detected with positron emission tomography imaging, cerebrospinal fluid samples, as well as a pTau217 blood test.^11,12^

Current treatments of AD focus on the earlier stages, making early detection crucial for effectively delaying disease progression. There is a growing demand for diagnostic tools and biomarkers for early detection, as early interventions improve the quality of life for AD patients.^13^

Idiopathic normal pressure hydrocephalus (iNPH) is a neurodegenerative disease characterized by the accumulation of cerebrospinal fluid in the brain’s ventricles. The current standard treatment is shunt surgery, which alleviates the symptoms, such as gait disturbances, urinary dysfunction, and cognitive deficits.^14^

The shunt treatment is most effective when performed as early as possible.^15^ If the shunt is installed at a later stage, its ability to improve cognitive deficits is limited. However, regarding motor symptoms, the shunt treatment tends to be more effective and its benefits last longer, although they eventually diminish over time.^14,15^

The challenge lies in determining whether a patient with iNPH also suffers from AD.^16^ The diseases often manifest simultaneously, and therefore share similar attributes, such as the amyloid and tau accumulations.^17,18^ The APOE ε4 gene allele is associated with beta amyloid plaque formation and increases the risk of AD, but does not predispose individuals to iNPH.^19^

There is also increasing research highlighting the possible role of neuroinflammation behind neurodegenerative disease pathogenesis. For instance, when the inflammatory markers from the cerebrospinal fluid were compared between healthy people and patients with iNPH, AD, mild cognitive impairment, and frontotemporal dementia, a study found differences in the cytokine levels, particularly in the iNPH group, compared with the other groups.^20^ Gastrointestinal dysbiosis and a epithelial damage, causing bacteria to translocate from the GI system, is known to promote systemic inflammation.^21^

In this study, we investigated whether the GM composition differs between healthy controls (CO) and AD patients, as well as between 2 neurodegenerative diseases, AD and iNPH. Comparing the GM differences between the AD and iNPH groups may help reveal novel candidates for differential diagnostic biomarkers.

## METHODS

### Research Ethics Statement

This study was approved by the Ethical Committee of Kuopio University Hospital (Dnro: 482/2017 and 276/2016) and adhered to the principles of the Declaration of Helsinki. Before participating, all study participants read the information letter and signed an informed consent.

### Study Design, Participants, and Protocol

We recruited 68 participants. The exclusion criteria included diabetes, depression, and other neurological disorders, such as Parkinson disease or severe dementia.

Cognitive performance of each participant was evaluated by the Consortium to Establish a Registry for Alzheimer Disease neuropsychological test battery (CERAD-NB), and the cognitive and functional status of the participant was assessed with the Clinical Dementia Rating scale (CDR).^22–24^ In addition, demographic interviews were conducted, as well as a clinical examination by a neurologist. The AD group consisted of 21 diagnosed mild AD cases, which were diagnosed by the revised National Institute on Aging and Alzheimer’s Association (NIA/AA) criteria.^25^

The cognitively healthy control group (CO, N=37) was recruited by the Brain Research Unit of the University of Eastern Finland. Study participants were classified as controls if their CDR score was 0.

The iNPH group was recruited from the NPH registry in Kuopio (http://www.uef.fi/nph). Ten individuals with shunt-treated iNPH were included, and they were the same study participants as described in our previous study.^7^ During the shunt surgery, AD-related pathology was excluded by obtaining biopsies from the right frontal cortex. Participants were clinically evaluated by a neurosurgeon, and they had no other neurodegenerative diseases or brain-related comorbidities.

To identify potential confounders in the GM composition, we collected data about the diet, diseases, and medications. Comorbidities, such as cardiovascular and metabolic diseases, were included in the adjusted statistical analysis.

Apolipoprotein E (ApoE) ε4 allele status was analyzed from venous blood samples using the QIAamp DNA Blood Mini Extraction kit (QIAGEN).^26^

### Fecal Sample Collection and DNA Analysis

Participants kept a food diary for 1 week before the fecal sample (FS) collection. FS were collected at home using containers prefilled with a DNA stabilizer (PSP Spin Stool DNA Plus Kit; STRATEC Molecular) and stored in home freezers until they were transported to the University of Eastern Finland in ice packages. The samples were then stored at the study site at −80°C. The sample collection was recommended to be done just before the scheduled study visit. During transportation to Helsinki for analysis, the samples were stored in dry ice.

The methods of DNA extraction and sequencing are described in more detail in our previous study.^7^ In summary, the DNA was extracted from the fecal samples using the PSP Spin Stool DNA Plus Kit by STRATEC Molecular. Following this, specific regions of the rRNA gene were amplified by Polymerase Chain Reaction and sequenced. The sequences were trimmed, and through the interference of amplicon sequence variants, the samples could be taxonomically classified.^27^ Potential contamination was assessed at various stages of the protocol.

### Statistical Analyses and Database Search

For statistical analyses of demographic data, we used IBM SPSS Statistics for Mac (Version 27.0, IBM, Armonk, NY). We analyzed the differences between groups for categorical variables, such as gender and ApoE allele status, using the χ^2^ test. For multiple comparisons of continuous variables (age, education, and CERAD-NB results), we used one-way ANOVA, followed by Bonferroni correction for post hoc group comparisons and for the post hoc comparisons.

The DNA sequencing information was analyzed using R software with a distance-based multivariate analysis method (permutational multivariate analysis of variance, as implemented in the vegan package) to evaluate the impact of clinical and technical variables on the sequencing data.^7,28,29^

On the basis of the results of the PERMANOVA models, we adjusted the models for differential abundance analysis as follows: for AD versus CO, the model was adjusted for arrhythmia and statin medication use. For AD versus iNPH, the model was adjusted for gender. The differential abundance analyses were performed in R software using the DESeq. 2 package.^30^

## RESULTS

### Study Population Demographics

The demographic data of the study groups is described in Table 1. In the AD group, the proportion of ApoE ε4 carriers (78.9%) was significantly higher compared with the CO group (37.8%) (*P*<0.005) and to the iNPH group (0%) (*P*<0.001).

**TABLE 1:** Clinical and Demographic Data of the Study Participants

	CO, N=37	AD, N=21	iNPH, N=10	*P*, CO-AD	*P*, CO-iNPH	*P*, AD-iNPH
Age, y	71.0 (5.1)	71.1 (6.9)	75.9 (5.7)	1.00	0.061	0.098
Education, y	12.6 (4.3)	12.5 (4.2)	10.0 (3.5)	1.00	0.261	0.354
Sex, female %	54 (20)	61.9 (13)	70 (7)	1.00	0.481	0.712
ApoE- ε4 allele carrier %	37.8 (14)	78.9(15)	0 (0)	**0.04**	**0.022**	**<0.001**
MMSE (30)	28.4 (1.5)	23.9 (3.0)	24.7 (3.6)	**<0.001**	**<0.001**	1.000

Values are presented as means and SDs, except for sex and ApoE ε4 carrier status, which are shown as percentages and the number of participants.

AD indicates Alzheimer disease; CO, control; iNPH, idiopathic normal pressure hydrocephalus; N, number of participants; M, mean. Bolded *P*-values=significant differences between groups (*P*≤0.05).

The MMSE score was significantly higher (*P*<0.001) in the CO group compared with the AD group (MMSE mean score 23.9±3.0). The score was also significantly higher (*P*<0.001) compared with the iNPH group (MMSE mean score 24.7±3.6). There was not a significant difference between the AD and iNPH groups. There were no statistically significant differences in age, sex, or years of education. We also analyzed comorbidities, medications and dietary habits, and found no significant differences between groups (data not shown).

### Microbiome Analysis

Results of the 16S amplicon-based analysis of GM are presented in Table 2. Compared with the CO group, the AD group showed a statistically significant increase (*P*_*adj*_ <0.001) in 7 bacterial genera. In the AD group, *Anaerovorax* genus and an unknown genus of the Comamonadaceae family were increased, and a decrease in *Enterobacter, Absicoccus, Buttiauxella, Raoultella*, and *Lacticaseibacillus* genera was detected.

**TABLE 2:** Significantly Different Bacterial Genera in the AD Group Compared With the CO and iNPH Groups

Genus	Group	Log2 fold change	*P* adj
	CO → AD		
* Anaerovorax*	**+**	2.42×10^−7^	0.00101
* *“*Unknown Genus 27”*	**+**	5.61×10^−8^	0.00101
* Enterobacter*	**−**	−2.95×10^−7^	1.66×10^−10^
* Absicoccus*	**−**	−1.91×10^−8^	0.00101
* Buttiauxella*	**−**	−3.78×10^−8^	0.000334
* Raoultella*	**−**	−6.13×10^−8^	5.07×10^−12^
* Lacticaseibacillus*	**−**	−9.32×10^−8^	7.18×10^−10^
	iNPH → AD		
* Paramuribaculum*	**+**	1.08×10^−6^	0.0285
* * *“Unknown Genus 16”*	**+**	2.28×10^−7^	4.60×10^−8^
* Ruficoccus*	**+**	2.12×10^−7^	6.83×10^−8^
* Mitsuokella*	**+**	1.47×10^−7^	0.00132
* Anaeromassilibacillus*	**−**	−3.73×10^−6^	0.0432
* Desulfovibrio*	**−**	−1.42×10^−8^	9.55×10^−7^

AD indicates Alzheimer disease; CO, control; iNPH, idiopathic normal pressure hydrocephalus; log2 fold change, effect size measured as fold change and expressed in logarithmic scale (base 2); P_adj_, adjusted *P*-value (*P*≤0.05); “Unknown Genus 27”, an unknown genus from the Comamonadaceae family; “Unknown Genus 16”, an unknown genus from the Desulfovibrionaceae family.

“+” indicates increased abundance in the second group relative to the first group; “−” indicates decreased abundance in the second group relative to the first group.

Compared with the iNPH group, we found 6 statistically significantly altered genera in the AD group (*P*_*adj*_<0.05). There was an increase in the *Paramuribaculum*, an unknown genus of Desulfovibrionaceae family, *Ruficoccus* and *Mitsuokella* genera, and a decrease in the *Anaeromassilibacillus* and *Desulfovibrio* genera.

## DISCUSSION

In this study, we analyzed the fecal samples from the CO, AD, and iNPH groups and compared the compositions of GMs. Our aim was to determine whether the GM composition differs in the AD group compared with both the CO and iNPH groups, and to explore whether fecal samples could serve as a potential source of biomarkers and a tool for evaluating intervention efficacy in AD and iNPH in the future. To our knowledge, our study is the first to demonstrate that the GM differs between the 2 neurodegenerative diseases. In the AD group, we identified 7 significantly altered genera compared with the CO group and 6 genera compared with the iNPH group.

In our study, the study participants in the AD group represented the mild stages of the disease (CDR 0.5 to 1.0) and they were all using AD-targeted symptomatic medication. All patients in the iNPH group represented pure iNPH-cases, they had undergone shunt surgery and none of them exhibited AD-like pathology or other neurodegenerative comorbidities based on brain biopsy and other clinical radiologic evaluations. However, we cannot exclude the possibility that both the shunt treatment and AD medication may have some effect on the GM.

### Differential Abundance Analysis

When comparing the AD group to the CO group, an increase in *Anaerovorax* and an unclassified genus of the Comamonadaceae family was detected. *Anaerovorax* consists of butyrate-producing bacteria.^31^ Butyrate is a short chained fatty acid known for its positive impact on the GI system, such as improving the epithelial barrier particularly in the context of a high-fiber Mediterranean diet.^32^ However, in another dietary fiber intervention study, the growth of *Anaerovorax* was reportedly inhibited.^33^ In addition, *Anaerovorax* has been associated with a high-fat, high-sugar diet.^34^ In contrast, the role of the Comamonadaceae family in human health remains unclear due to limited research. *Anaerovorax* is closely related to the Clostridium aminobutyricum species from the Clostridium genus, which has been reported to increase in AD patients.^31,35^

Among the decreased genera, *Absicoccus* and *Buttiauxella* are clinically of unknown importance. *Raoultella* is a genus, which may cause opportunistic infections.^36^ *Enterobacter* genus consists of opportunistic bacteria, some of which may exhibit antibiotic resistance abilities.^37^ These genera have not previously been linked to neurological diseases.

Clinically, the most notable finding is the decrease of *Lacticaseibacillus* in the AD group, a genus now classified separately from the *Lactobacillus* genus. The bacteria of this genus produce lactic acids, which are beneficial for health. For instance, in an intervention study, PD patients were given a supplement with a strain of *Lacticaseibacillus paracasei* and it was reported to alleviate nonmotor symptoms and constipation-related symptoms.^38^ This finding raises the question of whether a similar treatment could be effective in ameliorating AD symptoms, thereby encouraging further studies.

When comparing the GM composition between the iNPH and AD groups, we found 6 genera which were significantly altered in abundance. The bacterial genera that were increased in the AD group included *Paramuribaculum*, an unknown genus of the Desulfovibrionaceae family, *Ruficoccus* and *Mitsuokella*. We also detected a decrease in the *Anaeromassilibacillus* and *Desulfovibrio* genera.

The general relevance of most of these genera is currently unknown. A single study reported, that in novel HIV-patients, *Mitsuokella* was among the increased genera.^39^

Two genera decreased in the AD group, *Anaeromassilibacillus* and *Desulfovibrio. Anaeromassilibacillus* was associated with social anxiety disorder in one study.^40^ The *Desulfovibrio* genus has been linked with PD but the results are slightly conflicting.^4^

We have previously published the GM differences between the iNPH patients and the CO group.^7^ Interestingly, some of the genera found in that study are the same as in this current study. In the previous work, the amount of *Anaeromassilibacillus* was higher and the *Paramuribaculum* was lower in the iNPH group compared with the CO group. Here, we detected that in the AD group, the amount of *Paramuribaculum* genus was significantly higher, and the amount of *Anaeromassilibacillus* genus was significantly lower than in the iNPH group.

### ApoE ε4

In our study, the number of ApoE ε4 allele carriers was the highest in the AD group, as expected. APOE ε4 is an allele, which even carried as a heterozygote, increases the risk of sporadic AD. Other isoforms are APOE ε2, which may protect from AD and APOE ε3 with a neutral effect on the risk (Serrano-Pozo, Das and Hyman, 2021). It has previously been reported that iNPH carriers do not have a higher frequency of APOE e4 compared with cognitively normal participants (Pyykkö and colleagues, 2012). Our findings were in line with this result since none of the individuals of the iNPH group carried the APO e4 allele. This suggests that they are at a lower risk of having AD co-pathology

### Strengths and Limitations

The sample size in our study was slightly smaller compared with the Asian studies but similar in size, even slightly larger than in comparable Western study reports (Hung and colleagues, 2022). Further research with larger study populations may provide more insight into the matter. Also, we were not able to analyze bacterial diversity differences in this study due to limited research resources.

However, we were able to demonstrate significant differences between the study groups. Importantly, we showed that 2 different neurodegenerative diseases were associated with alterations in bacterial genera.

One of the strengths of this study was the establishment of clearly defined cohorts without overlapping pathologies. The ability to form 2 different groups, one consisting only of mild AD cases and the other group of only iNPH cases without AD pathology is notable, since the 2 diseases often manifest together.

Because our aim was to study specific differences in bacterial genera between the 2 neurodegenerative diseases, this focus may also be considered a limitation. In real-world clinical settings, older patients often present with mixed-type memory disorders.

As for our study demographics, there were no statistically significant differences between the groups, so the study population was quite homogenous. The biggest environmental factor affecting the composition of the GM is diet, and since the diet of the participants did not vary, we were able to avoid a significant confounding factor. The study participants were all recruited from the same geographical area in Finland, where the dietary preferences appear to be homogenous.

We aimed at ruling out potential confounding factors in advance by excluding participants with comorbidities, which potentially influence the GM composition or cognition, such as diabetes, other neurological diseases, or severe psychiatric disorders. Furthermore, we adjusted the statistical analysis for potential confounding factors, such as diet, medications, and other diseases, which came up in the demographic interviews.

A limitation, however, is that this was a cross-sectional study, and longitudinal studies would be encouraged to find out how the GM changes as AD progresses.

### Implications and Future Studies

Early diagnosis of AD is crucial for future interventions targeting the earliest stages of AD to effectively influence its progression. It would also be interesting to study how early the microbiome changes occur in the disease course before the cognitive deterioration. This encourages further research into whether the GM could provide tools for early detection and differential diagnostics of the neurodegenerative diseases in challenging cases.

The clinical significance of most of the bacterial genera mentioned in this study is still unknown. Basic research is needed in the field of microbiology to increase understanding of these bacteria. It is also interesting to study the effects of AD medications the GM composition as well as the effects of shunt treatment in the GM of an iNPH patient.

## CONCLUSIONS

We were able to distinguish differences in the gut bacteria in AD patients compared with cognitively healthy individuals and for the first time, also patients with iNPH. This provides us with new information to develop tools for differential diagnostics. The results encourage further studies to understand the role of GM in AD pathophysiology.
